# Surface Area to Volume Ratio of Electrospun Polydioxanone Templates Regulates the Adsorption of Soluble Proteins from Human Serum

**DOI:** 10.3390/bioengineering6030078

**Published:** 2019-08-31

**Authors:** Allison E. Fetz, Cristina A. Fantaziu, Richard A. Smith, Marko Z. Radic, Gary L. Bowlin

**Affiliations:** 1 Department of Biomedical Engineering, University of Memphis, Memphis, TN 38152, USA; 2 Department of Biomedical Engineering, College of Medicine, University of Tennessee Health Science Center, Memphis, TN 38163, USA; 3 Department of Microbiology, Immunology, and Biochemistry, College of Medicine, University of Tennessee Health Science Center, Memphis, TN 38163, USA

**Keywords:** electrospinning, electrospun template, tissue regeneration, biomaterials, protein adsorption, SAVR, neutrophils, neutrophil extracellular traps

## Abstract

Neutrophils, the first cells that interact with surface-adsorbed proteins on biomaterials, have been increasingly recognized as critical maestros in the foreign body response for guided tissue regeneration. Recent research has shown that small diameter (SD) fibers of electrospun tissue regeneration templates, which have a high surface area to volume ratio (SAVR), enhance the release of neutrophil extracellular traps (NETs) compared to large diameter (LD) fibers, resulting in impaired tissue regeneration. In this study, we evaluated the adsorption of eight human serum proteins on the surface of electrospun templates to investigate how protein adsorption may regulate the release of NETs. Electrospun polydioxanone templates made from SD fibers with high SAVR and LD fibers with low SAVR, were incubated with 0.2% human serum and in situ protein adsorption was quantified with infrared-based immunodetection. Of the detected proteins, IgM and vitronectin adsorbed at low levels, suggesting that they do not play a central role in the release of NETs. Contrastingly, albumin and IgG adsorbed rapidly to the surface of the templates. One-hundred to 200 times more IgG adsorbed on the templates compared to albumin, with significantly greater adsorption occurring on the SD templates with high SAVR. Given that neutrophils express receptors that interact with IgG during phagocytosis and NET release, these results suggest that SAVR-dependent adsorption of IgG on the SD electrospun templates may contribute to the up-regulated release of NETs. Overall, this study may aid in the design of immunomodulatory biomaterials that regulate NET release and thus the potential for neutrophil-driven tissue regeneration.

## 1. Introduction

The greatest challenge for the clinical translation of biomaterials in tissue regeneration applications is guiding integrative and functional tissue regeneration. The cellular and molecular events immediately following implantation establish the microenvironment that determines the biological performance of implants. During the immediate innate immune response, lipids, ions, sugars, and soluble proteins from the blood rapidly adsorb on the surface of the biomaterial to facilitate cell interactions with the biomaterial. The instantaneous and dynamic adsorption of protein on a material surface is known as the Vroman effect [1]. In general, smaller low-molecular weight proteins and those present at high concentrations adsorb first on the surface of a material. As time progresses, these proteins may be displaced by larger high-molecular weight proteins that have a greater affinity for the material surface. Material surface properties, such as hydrophobicity or hydrophilicity, charge, roughness, surface area, and chemistry, all govern protein adsorption and potential conformational changes while providing some selectivity [2]. Protein adsorption, in turn, influences the recruitment and attachment of vascular, stromal, and inflammatory cells over the course of several hours to orchestrate the foreign body response, which ultimately culminates in fibrous tissue encapsulation or elusive, functional tissue regeneration. While it is generally well-accepted that the outcome is largely dependent on the extent of the foreign body response, it is becoming increasingly recognized that the neutrophil and its interactions with the biomaterial play a central role in establishing the optimal microenvironment for functional tissue regeneration [3]. 

Neutrophils, the most abundant white blood cell in the blood, are the first dynamic line of defense interacting with surface-adsorbed proteins on the biomaterial within the first hour of the inflammatory response [4,5]. Through outside-in signaling via receptors on the cell surface, the diverse stimuli from the surface-adsorbed proteins are transduced into intracellular signals, ultimately resulting in a cellular response. Neutrophil responses can include phagocytosis, degranulation, and the generation of reactive oxygen species, which together create an intense attack that modulates the local microenvironment surrounding the biomaterial [6,7]. In addition to these well-described mechanisms of defense, neutrophils can also release neutrophil extracellular traps (NETs) through NETosis to trap and neutralize invading pathogens [8]. NETosis, a specialized form of antimicrobial cell death, results in the extrusion of a voluminous three-dimensional structure composed of decondensed chromatin, histones, various granule factors, and other antimicrobial proteins. While beneficial for combating infection, the dysregulated release of NETs is detrimental and associated with chronic inflammation, autoimmune disorders, tissue fibrosis, and thrombosis [9,10,11]. 

NETs are also released in response to various biomaterials, including titanium plates, nanoparticles, and electrospun templates [12,13,14]. Our group recently demonstrated that NETs were differentially released on the surface of electrospun polydioxanone (PDO) tissue regeneration templates [14]. Templates composed of small diameter (SD, 0.2–0.4 µm) fibers elicit a significantly greater release of NETs from interacting neutrophils compared to templates made of large diameter (LD, 1.0–3.0 µm) fibers, suggesting that the microenvironment created by the template architecture regulates the neutrophil response. More importantly, we also showed that the increased presence of NETs on the surface of the SD electrospun templates impaired tissue integration and promoted the development of a fibrotic capsule in vivo in a rat subcutaneous implant model [14]. Given that neutrophils interact with the surface of the biomaterial through adsorbed proteins, the protein constituents and their quantities on the electrospun template surfaces may be intimately linked to differential release of NETs, thereby playing a central role in establishing a microenvironment conducive to functional tissue regeneration. 

In this study, we evaluated the adsorption of eight proteins of interest: (1) albumin, (2) complement C1Q, (3) complement C3, (4) IgG, (5) IgM, (6) vitronectin, (7) fibrinogen, and (8) fibronectin on the surface of electrospun polydioxanone (PDO) templates. The purpose of the work was to characterize protein adsorption on the PDO templates in order to begin understanding how it may be linked to the differential engagement of neutrophil surface receptors, leading to strikingly different NET responses [14]. PDO templates were electrospun with SD and LD fibers to generate materials with high and low surface area to volume ratios (SAVR), respectively. We hypothesized that greater protein adsorption would occur on the SD templates with high SAVR. To replicate the culture conditions of our previous work, the templates were incubated with 0.2% human serum for different periods of time and protein adsorption on the templates was quantified with in situ infrared (IR)-based immunodetection to assess the effects of SAVR on protein adsorption. We found that SD templates with high SAVR adsorbed significantly more protein than LD templates with low SAVR. Furthermore, we determined that IgG adsorbs in the greatest quantity and most rapidly on the SD templates, suggesting that it may play a role in signaling NET release. Ultimately, understanding the constituents and dynamics of acute protein adsorption on electrospun tissue regeneration templates may provide insight into the potential mechanisms regulating NET release from neutrophils and their subsequent contribution to template-guided tissue regeneration. 

## 2. Materials and Methods 

### 2.1. Template Fabrication and Characterization

PDO (Bezwada Biomedical, LLC, Hillsborough, NJ, USA) was dissolved overnight in 1,1,1,3,3,3-hexafluoro-2-propanol (HFP, Oakwood Products, Inc., Estill, SC, USA) at 67 and 135 mg/mL to generate SD and LD templates, respectively. The solutions were loaded into a 5-mL syringe (Becton, Dickinson and Company, Pro. No. 309646, Franklin Lakes, NJ, USA) and attached to a 22.5-gauge (Becton, Dickinson and Company, Pro. No. 305156) and 18-gauge blunt needle (Becton, Dickinson, and Company, Pro. No. 305196) for SD and LD templates, respectively, that was connected to the positive lead of a Spellman CZE1000R power source (Spellman High Voltage Electronics Corp., Hauppauge, NY, USA). The syringe was placed on a syringe pump (Fisher Scientific, Model No. 78-01001, Pittsburgh, PA, USA), and the 67 and 135 mg/mL solutions were dispensed at 0.5 and 4 mL/h with an applied voltage of +11 and +25 kV, respectively. The fibers were collected over an airgap distance of 10 cm for the SD templates and 27.9 cm for the LD templates on a grounded stainless-steel cylindrical mandrel (2.5 cm diameter, 10 cm width) rotating at 1250 rpm and translating 6.5 cm/s over 13 cm. 

To generate scanning electron micrographs (SEMs), the templates were sputter coated (Electron Microscopy Sciences, EMS 550, Hatfield, PA, USA) with 5 nm of gold-palladium in an argon gas field and imaged with a FEI Nova NanoSEM^TM^ at +20 kV with a working distance of 5 mm and spot size of 3. The SEMs were then characterized to determine template fiber diameter using FibraQuant^TM^ 1.3 software (nanoScaffold Technologies, LLC, Chapel Hill, NC, USA) from a minimum of 250 semiautomated random measurements per image. The SAVR for 6-mm diameter punches of the SD and LD templates (*n* = 20) were then determined using Equations (1) and (2):

(1) $$Equivalent\;fiber\;length\;\left[cm\right]=\;\frac{mass\;of\;the\;template\;\left[g\right]}{material\;density\;\left[\frac{g}{cm^{3}}\right]*\pi *{\left(fiber\;radius\;\left[cm\right]\right)}^{2}},$$

(2) $$SAVR\;\left[\frac{m^{2}}{cm^{3}}\right]=\;\frac{Equivalent\;fiber\;length\;\left[m\right]*2*\pi *fiber\;radius\left[m\right]}{template\;volume\;\left[cm^{3}\right]},$$

The material density of PDO (Equation (1)) is 1.40 g/cm^3^, as determined by standard pycnometry with an Ultrapyc 1200e pycnometer (Quantachrome Instruments, Model MUPY-31, Boynton Beach, FL, USA). The volume of the templates (Equation (2)) was determined by measuring the thickness of the 6-mm diameter punches and multiplying it by the area of a 6-mm diameter circle to generate cylindrical volume.

### 2.2. IR-Based Immunodetection Assay and Validation

To quantify the adsorption of protein, we developed an IR-based immunodetection assay using standard curves and validated the method. First, a standard dilution for each protein (Table 1) was generated on Immobilon-FL PVDF membrane (EMD Millipore Corporation, Burlington, MA, USA) using a Bio-Dot Microfiltration Apparatus (BIO-RAD, Hercules, CA, USA) according to the manufacturer’s instructions. Briefly, 30 µL of protein (*n* = 3) diluted in Hank’s buffered salt solution (HBSS, Corning cellgro, Corning, NY, USA) was allowed to flow through the membrane by gravity for 45 min before applying the vacuum to draw through the remaining protein solution. The membranes were dried overnight before proceeding with processing and IR-based immunodetection.

Next, the standard dilutions were blocked with 5% non-fat milk (Nestle, Vevey, Switzerland) in PBS for 1 h at room temperature before overnight incubation with primary antibodies diluted in 5% milk at 4 °C (Table 2). Following, three 5-min washes with 0.1% Tween-20 in PBS were performed at room temperature with gentle agitation. The standard dilutions were then incubated with secondary antibodies conjugated to IR dyes (Table 2) diluted in 5% milk with 0.1% Tween-20 and 0.01% SDS for 1 h at room temperature to facilitate IR detection of adsorbed proteins. After two 5-min washes with 0.1% Tween-20 in PBS and a final 5-min wash with PBS, the standard dilutions were scanned on the 680 and 800 nm channels of the Odyssey CLx IR Imaging System (LICOR, Lincoln, NE, USA) with automatic intensity adjustment to generate fluorescence for each spot on the standard dilutions. Finally, using Image Studio version 5.x (LICOR), the relative fluorescent intensities of the spots were acquired by placing circular markers over the area of the spots. 

To validate the method, known amounts of each protein (*n* = 3) within the working ranges of the standard curves (Table 3) were applied to the PVDF membrane, dried overnight, and processed for IR-based immunodetection as described for the standard dilutions. Only four standard curves were validated based on data, indicating that four of the eight proteins of interest adsorbed on the electrospun templates, which is detailed below.

### 2.3. In Situ Protein Adsorption

For in situ protein adsorption, 6-mm diameter punches of the SD and LD templates (*n* = 6) were disinfected with a 30-min ethanol (Fisher Chemical, Cat. No. A407-1, Hampton, NH, USA) wash followed by three washes with 1x sterile HBSS for 10 min each. The disinfected templates were then placed in a 96-well cell culture plate and 150 µL of 0.2% normal pooled human serum (MP Biomedicals, LLC, Cat. No. 0823201, Solon, OH, USA) in HBSS or 150 µL of HBSS were added to the templates. The templates were incubated at 37 °C with 5% CO_2_ for 0.25, 0.5, 0.75, 1, 2, 3, and 6 h. After each time point, the serum solution was removed from the wells and discarded, and the templates were washed with 1x non-sterile phosphate buffered saline (PBS, HyClone) for 5 min with gentle agitation at room temperature to remove non-adsorbed protein. The templates were then fixed and stored in 10% buffered formalin at 4 °C overnight until analysis. 

### 2.4. IR-Based Immunodetection of Adsorbed Proteins on Templates

The adsorbed protein on the electrospun templates was quantified using the standard curves for each protein. First, free aldehyde groups on the fixed templates were quenched with three 5-min washes of 100 mM glycine in PBS with gentle agitation at room temperature. Then, the templates were processed for IR-based immunodetection as described for the standard curves. Each template was incubated with two primary and secondary antibodies to facilitate detection of two adsorbed proteins per template (Table 2). The relative fluorescence of the templates incubated with HBSS only was subtracted from the relative fluorescence of the templates incubated with serum to remove background fluorescence. Finally, protein adsorption was normalized to the mass of the 6-mm diameter punches.

### 2.5. Statistical Analysis

Data are presented as mean ± standard deviation. Fiber diameters and SAVRs were analyzed with a two-tailed t-test with Welch’s correction. For the standard curves, nonlinear regression was performed and goodness of fit was assessed with a replicates test for lack of fit. For validation of the IR-immunodetection assay, linear regression was performed and the coefficient of determination was calculated. An analysis of protein adsorption was executed with a two-way analysis of variance and Tukey multiple comparison procedure. All statistical analyses were performed in GraphPad Prism 6 at a significance level of 0.05.

## 3. Results

### 3.1. Electrospun Template Fiber Diameter Controls Surface Area

**Figure 1:**
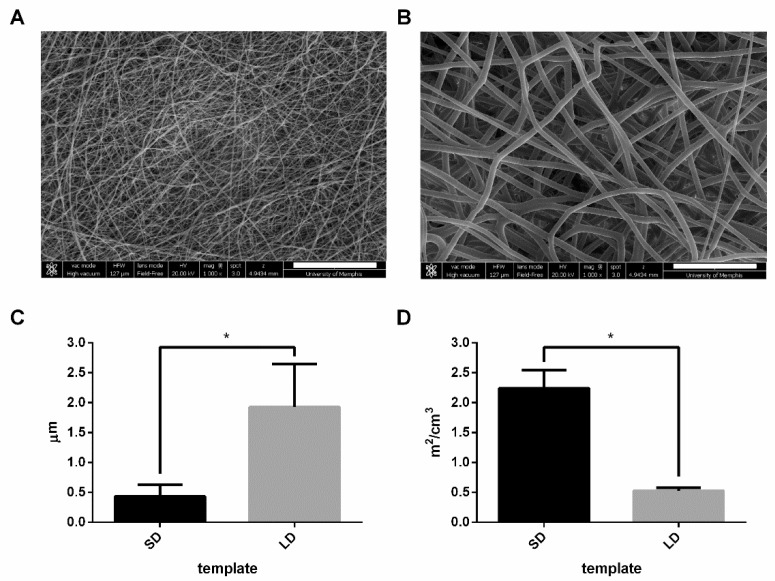
Electrospun small diameter (SD) and large diameter (LD) polydioxanone (PDO) templates. Representative scanning electron micrographs (SEMs) of (**A**) SD and (**B**) LD electrospun PDO templates (magnification = 1000×, scale bars = 30 µm). (**C**) The fiber diameter of the SD templates was significantly smaller than the LD templates which correlates to significantly greater (**D**) surface area to volume ratio (SAVR). The graphs show mean ± standard deviation from three independent experiments. * *p* < 0.05.

The low and high concentration PDO solutions resulted in electrospun templates with SD fibers and LD fibers, respectively (Figure 1A,B). The SD templates had significantly smaller (*p* < 0.05) fiber diameters, with an average of 0.43 ± 0.20 µm compared to the LD templates with fiber diameters of 1.93 ± 0.72 µm (Figure 1C). The fiber diameters of the SD templates correlated to an average SAVR of 2.07 ± 0.33 m^2^/cm^3^, which was significantly greater (*p* < 0.05) than the average SAVR for the LD templates of 0.55 ± 0.07 m^2^/cm^3^ (Figure 1D). Since the SD templates have nearly four times the surface area per volume, these data indicate that the SD templates have significantly more surface area available for protein adsorption. 

### 3.2. IR-based Immunodetection Quantifies Protein Adsorption

**Figure 2:**
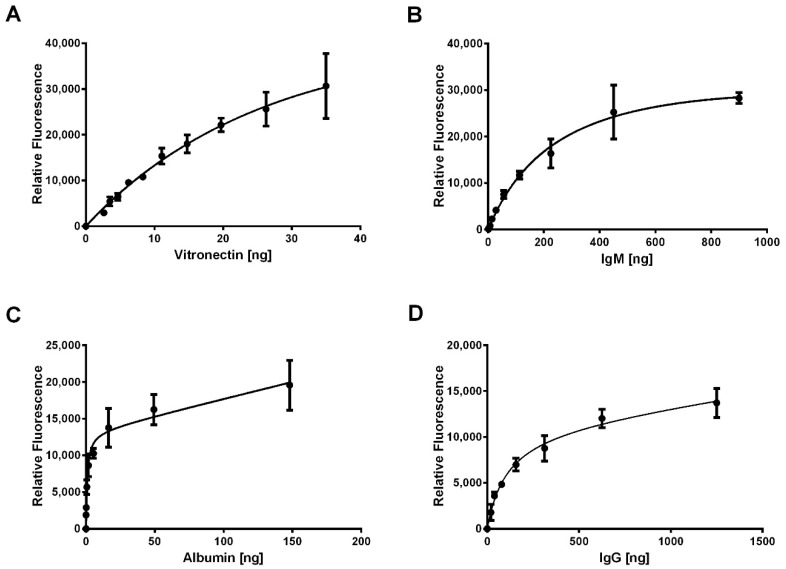
Standard curves generated from IR-based immunodetection. Dilutions of (**A**) vitronectin, (**B**) IgM, (**C**) albumin, and (**D**) IgG were vacuum blotted onto PVDF membranes, dried, and processed for detection with IR-based immunodetection. Each point in the standard curves was generated from three replicates. Error bars represent standard deviation.

For each protein, a standard curve was created to quantify adsorption through IR-based immunodetection. Figure 2 shows the standard curves with the nonlinear regression for vitronectin, IgM, albumin, and IgG. There was no adsorption of complement C1Q, complement C3, fibrinogen, and fibronectin detected on the electrospun templates, so these standard curves are not shown. For vitronectin (Figure 2A), the nonlinear regression (Equation (3)) produced a well-fitting curve with no evidence of an inadequate model from the lack of fit test (p = 0.99). Similar results were obtained for IgM (Figure 2B, Equation (4), *p* = 0.98), albumin (Figure 2C, Equation (5), *p* = 0.62) and IgG (Figure 2D, Equation (6), *p* = 0.65).

(3) $$y=\;\frac{115003x}{\left(57.53\;+\;x\right)}-373.6x\;\;y\;=\;\mathrm{relative}\;\mathrm{fluorescence},\;x\;=\;\mathrm{ng}\;\mathrm{of}\;\mathrm{vitronectin},$$

(4) $$y=\;\frac{43725x}{\left(292.1\;+\;x\right)}-5.052x\;\;y\;=\;\mathrm{relative}\;\mathrm{fluorescence},\;x\;=\;\mathrm{ng}\;\mathrm{of}\;\mathrm{IgM},$$

(5) $$y=\;\frac{13200x}{\left(0.89\;+\;x\right)}+45.83x\;\;y\;=\;\mathrm{relative}\;\mathrm{fluorescence},\;x\;=\;\mathrm{ng}\;\mathrm{of}\;\mathrm{albumin}\;,$$

(6) $$y=\;\frac{11247x}{\left(107.2\;+\;x\right)}+2.84x\;\;y\;=\;\mathrm{relative}\;\mathrm{fluorescence},\;x\;=\;\mathrm{ng}\;\mathrm{of}\;\mathrm{IgG}\;,$$

**Figure 3:**
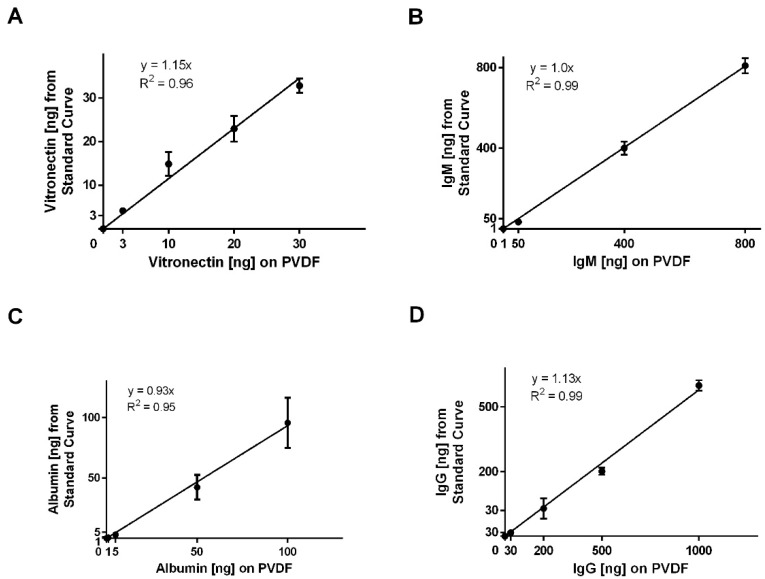
Validation of the IR-based immunodetection assay. The adsorptions of known amounts of (**A**) vitronectin, (**B**) IgM, (**C**) albumin, and (**D**) IgG were plotted against the interpolated values from the standard curves. Each point was generated from three replicates. The error bars represent standard deviation.

Additionally, the assay was validated for albumin, IgG, IgM, and vitronectin (Figure 3) by adsorbing known amounts of each protein to the PVDF membranes and assessing the interpolation from the standard curves. For all four proteins, the linear regressions for adsorbed protein versus the interpolated value from the standard curves had slopes near 1.0 and high coefficients of determination, indicating that assay accurately quantified the mass of the adsorbed proteins. Together, these standard curves were used to quantify adsorption of vitronectin, IgM, IgG, and albumin on the templates based on relative IR fluorescence.

### 3.3. SAVR Regulates In Situ Protein Adsorption

**Figure 4:**
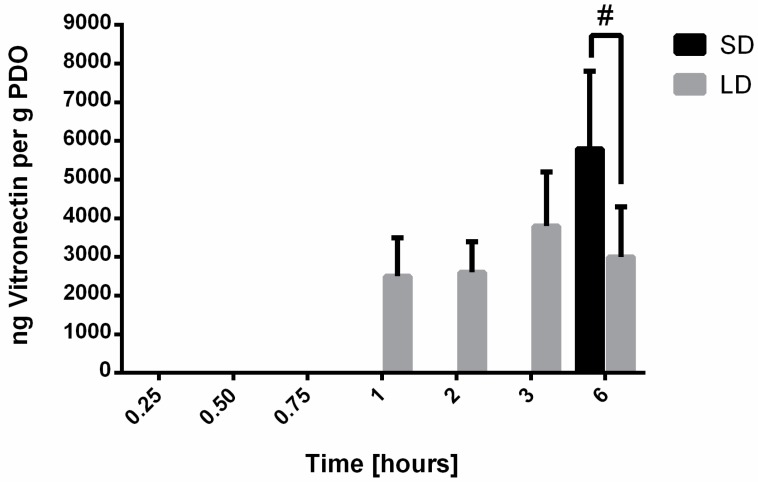
Vitronectin adsorption on electrospun PDO templates. Data shown as mean ± standard deviation from three independent experiments; # significant difference between SD and LD templates (*p* < 0.05).

**Figure 5:**
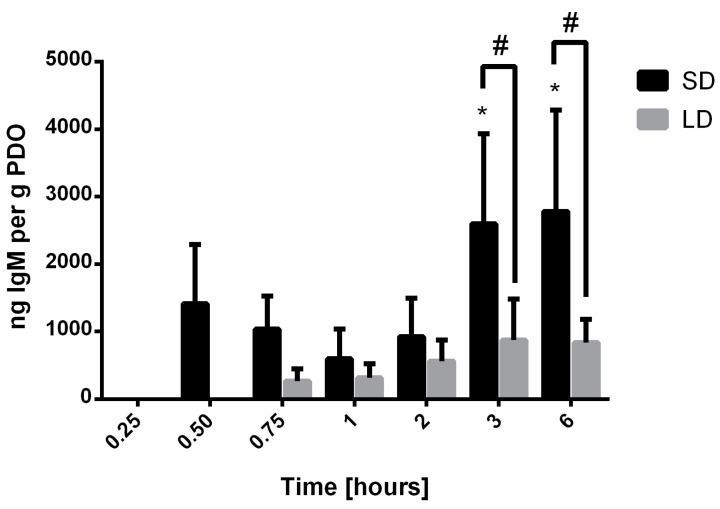
IgM adsorption on electrospun PDO templates. Data shown as mean ± standard deviation from three independent experiments; # significant difference between SD and LD templates (*p* < 0.05), * significant difference from 0.25 through 2-h time points for SD (*p* < 0.05).

**Figure 6:**
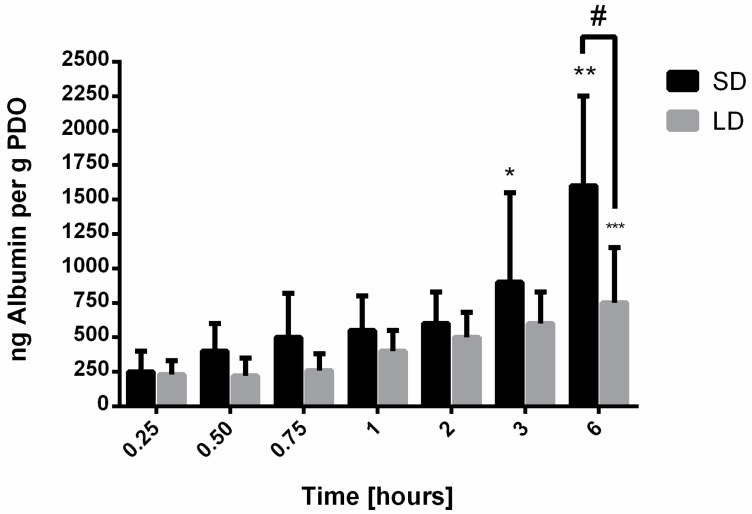
Albumin adsorption on electrospun PDO templates. Data shown as mean ± standard deviation from three independent experiments; * significant difference from 0.25, 0.50, and 0.75-h time points (*p* < 0.05), ** significant difference from all other time points for SD templates (*p* < 0.05), *** significant difference from 0.25 through 1-h time points for LD templates (*p* < 0.05), # significant difference between SD and LD templates (*p* < 0.05).

**Figure 7:**
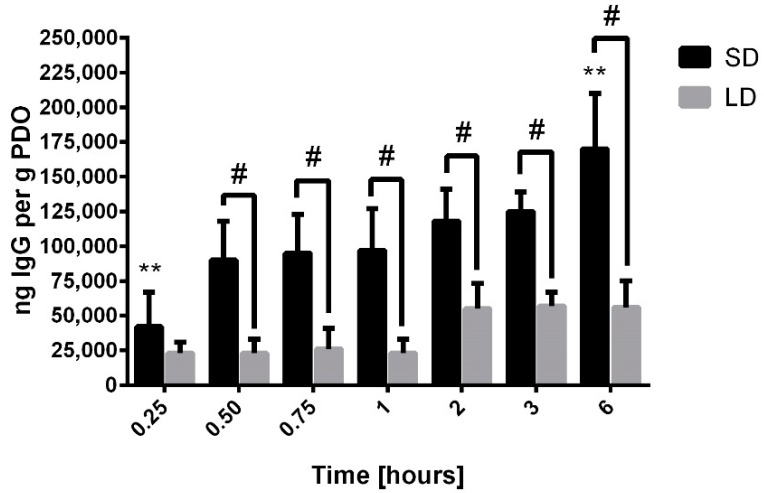
IgG adsorption on electrospun PDO templates. Data shown as mean ± standard deviation from three independent experiments; ** significant difference from all other time points for SD templates (*p* < 0.05), # significant difference between SD and LD templates (*p* < 0.05).

In order to highlight the differences in adsorption due to SAVR, the mass of adsorbed protein was normalized to mass of the 6-mm diameter template punches. Figure 4, Figure 5, Figure 6 and Figure 7 show the results from the IR-based quantification of the in situ adsorption of vitronectin, IgM, albumin, and IgG. There was no detection of complement C3, fibronectin, or fibrinogen. Notably, the Odyssey CLx Imaging System utilizes two IR lasers that penetrate through three-dimensional structures. This facilitates the full-thickness quantification of protein adsorption on the complex surface geometry of the electrospun templates. 

For vitronectin (Figure 4), adsorption was not detected until the 1-h time point for the LD templates and the 6-h time point for the SD templates. Upon adsorbing on the LD templates, the mass of vitronectin did not change over time. Despite the delay on SD templates, the amount of vitronectin adsorption at 6 h was significantly greater (*p* < 0.05) than on the LD templates, indicating that the high SAVR of the SD templates facilitated greater protein adsorption. Nonetheless, the adsorption of vitronectin on both templates was not detectable until at least 1 h after exposure to serum. In the physiological environment, neutrophils have already swarmed to the site of inflammation, clustered around the biomaterial, and began interacting and responding within the first hour, which suggests that vitronectin adsorption may not play an integral role in the initial template-induced release of NETs [5].

Figure 5 shows the results from the IR-based quantification of IgM adsorption. The adsorption of IgM was not detected until the 0.50-h time point for the SD templates and the 0.75-h time point for the LD templates. Adsorption on the SD templates initially decreased between 0.50 h and 1 h and then gradually increased between the 2-h and 6-h time points, suggesting that the dynamics of adsorption are temporal. At all the time points, the adsorption of IgM was greater on the SD templates, and by 3 h, it was significantly greater (*p* < 0.05) on the SD templates compared to the LD templates. These results indicate that the high SAVR of the SD templates promote greater protein adsorption compared to the LD templates. Nonetheless, the relatively low levels of IgM adsorption, similar to those for vitronectin, suggest that IgM does not significantly contribute to the template-induced release of NETs.

In contrast to vitronectin and IgM, albumin (Figure 6) was detected on the SD and LD templates at all time points and increased significantly over time for both templates. From 0.25 h to 6 h, there was greater adsorption on the SD templates compared to the LD templates, and by 6 h, albumin adsorption was significantly greater on the SD templates compared to the LD templates (*p* < 0.05). While increasing on both, the rate of albumin adsorption on the SD templates was nearly two times greater, with an average increase of 270 ng per hour, compared to the LD templates, which increased on average by 110 ng per hour. These data suggest that the high SAVR of the SD templates accelerated the adsorption of albumin and resulted in greater total adsorption over time. Since it was detected at all time points, it is possible that the adsorption of albumin contributes to the regulation of the neutrophil response. 

Similarly to albumin, IgG (Figure 7) was detected on the SD and LD templates at all time points. However, by half an hour, the adsorption of IgG was significantly greater on the SD templates compared to the LD templates (*p* < 0.05). Though it increased significantly on the SD templates, IgG adsorption did not significantly increase over time on the LD templates. The average rate of adsorption on the SD templates was 20,200 ng per hour compared to 5400 ng per hour for the LD templates. The strikingly different rates of adsorption resulted in nearly four times greater IgG adsorption on the SD templates by half an hour. These results suggest that significantly more IgG is adsorbed on the SD templates in the critical first hour after exposure to serum, which may contribute to the contrasting cell responses to SD and LD templates. Furthermore, compared to albumin, IgG adsorption on the SD and LD templates over the first hour is 200-times greater and 100-times greater, respectively. Based on the high magnitude of IgG adsorption, these data indicate that IgG may play a significant role in directing the differential release of NETs in response to electrospun polymeric biomaterials.

**Figure 8:**
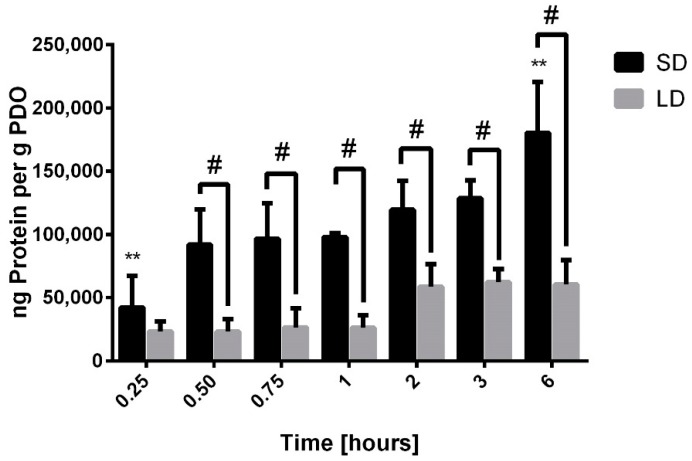
Total protein adsorption on electrospun PDO templates. The graph shows mean ± standard deviation from three independent experiments. ** indicates a significant difference from all the other time points for SD templates (*p* < 0.05) # indicates a significant difference between SD and LD templates (*p* < 0.05).

Finally, Figure 8 shows the total protein adsorption on the templates based on the detectable and quantifiable adsorption of vitronectin, IgM, albumin, and IgG. For all the time points, total protein adsorption was greater on the SD templates compared to the LD templates, and by half an hour, the difference was significant (*p* < 0.05). While the average rate of adsorption on the LD templates was 14,000 ng per hour, the average rate of adsorption on the SD templates was 40,000 ng per hour. These data are dominated by the adsorption of IgG and suggest that in general, the high SAVR of the SD templates facilitates greater protein adsorption compared to the LD templates. Taken together, the IR-based immunodetection of our eight proteins of interest indicates that the significant adsorption of IgG on the surface of the electrospun SD templates is driven by SAVR, which may orchestrate the outside-in signaling to stimulate NET release from interacting neutrophils.

## 4. Discussion

The neutrophil is increasingly recognized as a dynamic central player in the foreign body response to a biomaterial. Recent work examining neutrophil interactions with electrospun tissue regeneration templates has shown that the architecture of the electrospun templates differentially regulates the release of NETs and that an abundance of NETs impairs tissue regeneration in vivo [14]. Since neutrophils represent the first line of defense interacting with and responding to an electrospun template, it is expected that their responses, including NET release, have significant implications in the microenvironment modulation around a biomaterial and the progression of template-guided in situ tissue regeneration. In this study, we aimed to characterize protein adsorption on SD and LD electrospun templates to begin understanding if protein adsorption drives the differential engagement of neutrophil receptors to regulate NET release.

Electrospun PDO templates were fabricated from low and high concentration solutions to generate SD and LD templates with significantly different fiber diameters and SAVRs. In order to make comparisons, the SD and LD templates for this study were fabricated with the same average fiber diameters of the templates used to evaluate NET release in our previous work [14]. The SD templates had a fiber diameter that was nearly 4-times smaller than the LD templates, correlating to a SAVR that was nearly 4-times greater. Therefore, the significantly smaller fiber diameter of the SD templates provided more surface area for the potential adsorption of protein per unit mass of the electrospun templates. 

Using an IR-based immunodetection, which was validated for protein quantification, we examined the adsorption of eight proteins: (1) albumin, (2) complement C1Q, (3) complement C3, (4) IgG, (5) IgM, (6) vitronectin, (7) fibrinogen, and (8) fibronectin. While other proteins are present in the serum, which may be adsorbed to the templates, it is not feasible to study all protein constituents, and these eight proteins were selected for their high concentrations in serum and important roles in innate immunity. To replicate the methods from our previous experiments, we incubated the templates with commercially available 0.2% serum in HBSS that is produced by clotting [14]. Since our aim was to begin understanding how protein adsorption was regulated by SAVR and its potential implications in NET release, we did not quantify adsorption with purified protein solutions. Purified protein solutions would likely exhibit different adsorption profiles for each of the eight proteins, given that the Vroman effect is partly dependent on which proteins are present and their concentrations. Using purified solutions would undermine the complexity of an in vitro and in vivo microenvironment with a milieu of proteins [15]. Additionally, the concentration of each protein in the serum was not quantified before the experiments because our goal was not to determine what percentage of available protein adsorbs to the templates. However, we did verify that the total protein concentration for the serum fell within the normal range of 6.0–8.0 g/dL [16]. 

The results indicate that there was no detectable adsorption of complement C3, complement C1Q, fibrinogen, or fibronectin on the SD and LD templates. Since the experiment utilized 0.2% serum, which is depleted of most fibrinogen and fibronectin during coagulation, a lack of fibrinogen and fibronectin adsorption onto the templates was not surprising [17]. In addition, neither complement C3 nor complement C1Q adsorption was detected on the SD or LD templates. Despite their significant roles in innate immunity, the lack of complement adsorption may be due to their low affinity for the material surface and their relatively low concentrations in serum, which cannot compete with the high concentrations of other proteins such as albumin and IgG [16,18,19,20]. For vitronectin, IgM, albumin, and IgG, the results indicate that SAVR regulated protein adsorption and that the high surface area of the SD templates adsorbed significantly more protein compared to the LD templates with low surface areas. These results were anticipated, since it is established that surface area regulates protein adsorption on biomaterial surfaces. Furthermore, no significant desorption of these proteins was observed over the 6-h study, indicating that their affinity for the material surface was large enough to prevent competitive displacement by other high molecular weight proteins in this specified time frame. 

Vitronectin, a low molecular weight glycoprotein, plays a role in cell adhesion and spreading. With regards to the neutrophil, it has been shown to modulate adhesion, contribute to proinflammatory processes, and inhibit neutrophil apoptosis through integrin-dependent signaling pathways [21,22,23]. The adsorption of vitronectin to the electrospun templates was delayed until 1 h after exposure to serum on the LD templates and 6 h after exposure to serum on the SD templates. In addition, the magnitude of adsorption was lower compared to other detected proteins. Given this information, the adsorption of vitronectin on the electrospun templates likely does not regulate template-induced NETosis but may influence neutrophil viability and thus their ability to modulate the microenvironment through other cell responses such as degranulation and the secretion of cytokines and chemokines [6,7]. 

Similarly to vitronectin, the adsorption of IgM was delayed until 0.50 h after exposure to the serum for the SD templates and 0.75 h after exposure to the serum for the LD templates with relatively low levels of adsorption occurring on both the SD and LD templates. Compared to other immunoglobulins, IgM only accounts for 10–15% of the antibodies present in the serum, which, together with its affinity for PDO, may explain the delayed low levels of adsorption [24]. Even though IgM can play a central role in complement activation and thus the neutrophil response to the biomaterial, our data suggest that it is likely not a central player in the regulation of NET release on the surface of the electrospun templates [18]. 

Contrasting to vitronectin and IgM, both albumin and IgG adsorbed rapidly and readily to the surface of the SD and LD templates. Albumin adsorption increased over time on the templates and was significantly greater on the SD templates, suggesting that the high SAVR facilitated greater protein adsorption. As a non-adhesive protein that plays an integral role in maintaining oncotic pressure, albumin has been reported to work synergistically with adhesive proteins to regulate cell adhesion and spreading by exposing binding sites on the adhesive proteins [25]. In the case of neutrophil interactions, albumin has been shown to promote adhesion but prevent spreading and the generation of hydrogen peroxide [26]. Recently, Neubert et al. showed that albumin actually inhibits NET release when neutrophils are treated with pharmacological stimuli and cultured in the presence of albumin [27]. Therefore, although significantly more albumin adsorbed to the surface of the SD templates, these data suggest that albumin does not play a direct role in regulating NET release and another dominant protein on the surface of the material may be responsible for stimulating NETosis. 

In fact, compared to albumin, 100- to 200-times more IgG adsorbed on the surface of the electrospun templates over the first hour, suggesting that the greatest proportion of the surface area was covered by IgG. These results are not surprising because IgG is the second most abundant protein in serum, present at a concentration of 11 mg/mL, and is the most abundant immunoglobulin in circulation [28]. The absorption was also significantly greater on the surface of the SD templates by half an hour, and the rate of adsorption over the 6-h study was four times greater on the SD templates compared to the LD templates. These data indicate that IgG is the dominant protein on the surface of the SD templates, potentially regulating the outside-in signaling, leading to the differential release of NETs. 

We are not the first to quantify IgG adsorption on the surface of biomaterials. IgG has previously been shown to adsorb readily to the surface of biomaterials through strong hydrophobic interactions that can alter its secondary structure [2]. These strong hydrophobic interactions inhibit its desorption from the surface of a material, which is consistent with previous work showing that the dynamics of the Vroman effect are not as prominent on hydrophobic surfaces like our PDO templates [29,30]. On the surface of hydrophobic Teflon, Vermeer et al. found that IgG adsorbs through denaturation of the Fab fragments, leaving the Fc fragment unperturbed for potential recognition by cell receptors and outside-in signaling [31]. 

The neutrophil expresses two types of receptors for the Fc domain of IgG (FcγR), which play a major role in mediating phagocytosis and the secretion of inflammatory mediators [32]. In resting neutrophils, FcγRIIIb binds most of the ligand on the surface of the antibody-coated substrate [33]. However, when a neutrophil is activated during inflammation, a high number of strongly signaling FcγRIIa receptors engage the IgG-covered target, working synergistically with FcγRIIIb to initiate inflammatory signaling [34]. Traditionally, this signaling promotes the phagocytosis of the noxious stimuli, but recent work has shown that the opsonization of beads with IgG promotes a rapid increase in NET release that functions collaboratively with phagocytosis [35]. It is plausible that since our electrospun materials cannot be phagocytosed, all of the signals generated by FcγR ligation are transduced into rapid and abundant NET release on the IgG-coated SD templates. Moreover, recent work studying Toll-like receptor (TLR)-FcγR crosstalk in neutrophils has shown that the engagement of TLR7/8 by soluble immune complexes induced shedding of FcγRIIa [36]. The shedding of FcγRIIa reduces the phagocytic capacity of neutrophils and shifts the signaling toward NETosis. Together, these studies support our finding that the SAVR-dependent adsorption of IgG may be the major polarizing event occurring on electrospun templates, resulting in the engagement of cell receptors that up-regulate the release of NETs on SD templates. 

In order to determine whether IgG adsorption is intimately linked to biomaterial-induced NETosis, future work will include investigating how IgG adsorbs to the electrospun PDO templates and if the adsorption induces conformational changes. In addition, modulating the composition of the serum or inhibiting the activity of the FcγRs on the neutrophils will be critical for linking receptor ligation to NET release. Upon understanding the role of IgG, the surface properties of the electrospun templates can be modulated to regulate adsorption and possible induced conformational changes in the initial critical hours of the acute inflammatory response. In summary, we showed that the adsorption of protein from human serum was regulated by electrospun template SAVR and that the high surface area of the SD templates significantly increased protein adsorption. Furthermore, we found that IgG adsorbs abundantly to the SD templates, which may drive outside-in signaling to up-regulate the release of NETs, a topic of further investigation. Due to the increasingly acknowledged role of the neutrophil in functional tissue regeneration, this work provides a foundation for the future development of biomaterials that harness the dynamic responses of the neutrophil to promote wound healing and accelerate their translation into the clinical setting. 

## 5. Conclusions

The release of NETs on the surface of electrospun tissue regeneration templates may function as a significant preconditioning event that orchestrates the progression of the foreign body response and the potential for functional tissue regeneration. Recent research has shown that SD templates, which have high SAVR, up-regulate the release of NETs compared to LD fibers, resulting in impaired tissue regeneration. In this study, we found that SD templates significantly increased the adsorption of soluble serum protein. Additionally, we showed that IgG adsorbed rapidly on the surface of SD templates and at the greatest quantity compared to other proteins. Given the role of IgG in innate immunity and the release of NETs, these findings suggest that IgG may be linked to the differential modulation of NETosis on electrospun templates as a function of SAVR. Ultimately, understanding how protein adsorption on electrospun templates regulates neutrophil interactions will enhance the design of immunomodulatory biomaterials that utilize the neutrophil as an initiator and driver of biomaterial-guided tissue regeneration. 

## Figures and Tables

**Table 1:** Each protein was spotted onto a PVDF membrane to create standard curves for quantification of protein adsorption. Each point in the standard curves was produced from three replicates.

Protein	Upper Limit of Standard Curve [ng]	Lower Limit of Standard Curve [ng]	Dilution Factor
Human albumin (Novus Biologicals, Cat. No. NBP2-47623)	148	0.07	3
Human complement C1Q (Millipore Sigma, Cat. No. 2048761MG)	100	17.8	3/4
Human complement C3b (Millipore Sigma, Cat. No. 204860)	600	4.7	2
Human fibrinogen (Invitrogen, Prod. No. RP-43142)	800	4.1	2/3
Human fibronectin (ThermoFisher Scientific, Cat. No. 33016015)	150	20.0	3/4
Human IgG (Invitrogen, Prod. No. 31154)	1250	19.5	2
Human IgM (Millipore Sigma, Cat. No. 4017991MG)	900	0.11	2
Human vitronectin (Life Technologies, Cat. No. PHE0011)	35	2.6	3/4

**Table 2:** Primary antibodies were paired for immunodetection of two adsorbed proteins per template via two-color IR detection. The paired primary antibodies were from different species while the secondary antibodies were from the same species.

Paired Protein Detection	Primary Antibody	Dilution	Secondary Antibody	Dilution
Albumin and IgG	Mouse anti-albumin (Abcam ab10241)	1:2000	IRDye 680LT goat anti-mouse (LICOR, Part no. 926-68052)	1:20,000
	Rabbit anti-IgG (Abcam ab109489)	1:2000	IRDye 800CW goat anti-rabbit (LICOR, Part no. 925-32211)	1:20,000
Complement C3 and Fibronectin	Mouse anti-complement C3 (Abcam ab11871)	1:1000	IRDye 680RD goat anti-mouse (LICOR, Part no. 925-68070)	1:20,000
	Rabbit anti-fibronectin (Abcam ab32419)	1:1000	IRDye 800CW goat anti-rabbit (LICOR, Part no. 925-32211)	1:20,000
Complement C1Q and IgM	Mouse anti-complement C1Q (Invitrogen MA1-83963)	1:2000	IRDye 680RD goat anti-mouse (LICOR, Part no. 925-68070)	1:20,000
	Rabbit anti-IgM (Abcam ab212201)	1:1000	IRDye 800CW goat anti-rabbit (LICOR, Part no. 925-32211)	1:20,000
Vitronectin and Fibrinogen α chain	Mouse anti-vitronectin (Abcam ab13413)	1:600	IRDye 680LT goat anti-mouse (LICOR, Part no. 926-68050)	1:20,000
	Rabbit anti-fibrinogen α chain (Abcam ab92572)	1:2000	IRDye 800CW goat anti-rabbit (LICOR, Part no. 925-32211)	1:20,000

**Table 3:** Known amounts of each protein were adsorbed to PVDF to validate the standard curves. Each point was produced from three replicates.

Protein	Point 1 [ng]	Point 2 [ng]	Point 3 [ng]	Point 4 [ng]	Point 5 [ng]
Albumin	0	1	5	50	100
IgG	0	30	200	500	1000
IgM	0	1	50	400	800
Vitronectin	0	3	10	20	30
